# Improving implementation of evidence-based practice in mental health service delivery: protocol for a cluster randomised quasi-experimental investigation of staff-focused values interventions

**DOI:** 10.1186/1748-5908-8-75

**Published:** 2013-07-02

**Authors:** Virginia Williams, Lindsay G Oades, Frank P Deane, Trevor P Crowe, Joseph Ciarrochi, Retta Andresen

**Affiliations:** 1 School of Psychology, University of Wollongong, Wollongong, Australia; 2 Australian Institute of Business Wellbeing, Sydney Business School, University of Wollongong, Wollongong, Australia; 3 Illawarra Institute for Mental Health, University of Wollongong, Wollongong, Australia; 4 School of Psychology, University of Wollongong, Wollongong, Australia; 5 School of Social Sciences and Psychology, University of Western Sydney, Sydney, Australia; 6 Illawarra Institute for Mental Health, University of Wollongong, Wollongong, Australia

**Keywords:** Values clarification, Autonomous motivation, Transfer of training

## Abstract

**Background:**

There is growing acceptance that optimal service provision for individuals with severe and recurrent mental illness requires a complementary focus on medical recovery (*i*.*e*., symptom management and general functioning) and personal recovery (*i*.*e*., having a ‘life worth living’). Despite significant research attention and policy-level support, the translation of this vision of healthcare into changed workplace practice continues to elude. Over the past decade, evidence-based training interventions that seek to enhance the knowledge, attitudes, and skills of staff working in the mental health field have been implemented as a primary redress strategy. However, a large body of multi-disciplinary research indicates disappointing rates of training transfer. There is an absence of empirical research that investigates the importance of worker-motivation in the uptake of desired workplace change initiatives. ‘Autonomy’ is acknowledged as important to human effectiveness and as a correlate of workplace variables like productivity, and wellbeing. To our knowledge, there have been no studies that investigate purposeful and structured use of values-based interventions to facilitate increased autonomy as a means of promoting enhanced implementation of workplace change.

**Methods:**

This study involves 200 mental health workers across 22 worksites within five community-managed organisations in three Australian states. It involves cluster-randomisation of participants within organisation, by work site, to the experimental (values) condition, or the control (implementation). Both conditions receive two days of training focusing on an evidence-based framework of mental health service delivery. The experimental group receives a third day of values-focused intervention and 12 months of values-focused coaching. Well-validated self-report measures are used to explore variables related to values concordance, autonomy, and self-reported implementation success. Audits of work files and staff work samples are reviewed for each condition to determine the impact of implementation. Self-determination theory and theories of organisational change are used to interpret the data.

**Discussion:**

The research adds to the current knowledge base related to worker motivation and uptake of workplace practice. It describes a structured protocol that aims to enhance worker autonomy for imposed workplace practices. The research will inform how best to measure and conceptualise transfer. These findings will apply particularly to contexts where individuals are not ‘volunteers’ in requisite change processes.

**Trial registration:**

ACTRN: ACTRN12613000353796.

## Background

Provision of evidence-based services has been a priority in mental health systems in English speaking mental health services for over a decade. This has been driven by a growing awareness and concern that service delivery does not always reflect what is known to be best practice [1]. In response, policy makers have sought ways to narrow this gap and support the translation of research into practice. Within the mental health field, this has included explication of ‘recovery’ as a specific priority both at the policy level and within charters that encompass mental health organisations [2-4]. Recovery can be defined as ‘a way of living a satisfying, hopeful and contributing life’ beyond the limiting effects of mental illness [5].

Worldwide, health systems and the service providers have made significant efforts to re-define programmes and develop staff in alignment with the recovery philosophy and evidence base in order to enhance capacity and further decrease the research-practice gap [6]. The Collaborative Recovery Model (CRM) [7] is one approach that includes evidence-based intervention components, including those that focus on strengths, values, and goal striving [5,8,9]. Despite this, the literature contains numerous examples of disappointing attempts to implement evidence into mental healthcare *e*.*g*., [3,10] and healthcare more broadly [11,12].

Scientific evidence that something is ‘best practice’ is not adequately persuasive in changing the behaviour of staff [13,14]. Translating and using research in practice is a complex process impacting at various organisational levels, including individual staff. Recent enhancements to models of health system change acknowledge the key role of the staff or ‘local actors’ among the numerous contextual and innovation-specific factors [15,16]. Practitioners and managers are not passively persuaded by new practices even when the evidence to support them is sound. Instead, decisions made by managers and practitioners are based on a number of individual factors such as personal experience, clinical judgment, inference, intuition, and advice [6]. People do not implement because of a rational consideration of the evidence alone. Motivation emerges as a key factor [17].

More specifically, the literature on health behaviour change provides a foundation for understanding how to change work behaviours. It is well established within the health behaviour change literature that knowing there is ‘good evidence’ for the benefits of a specific change is a poor predictor of changed behaviour [18]. People are most highly motivated to change when the desired behaviour is something that aligns with their beliefs and values [19]. While this is true for individuals generally, it is likely to be even more salient in professions where individuals are drawn to the work for values-based reasons [20,21]. In a profession where values are central, connecting staff to the ethos in which the change is embedded is likely to be highly important to the promotion of uptake. We argue that values are persuasive in motivating staff to change their practice. In this context, we now discuss values as a key aspect of motivation.

Motivation for change is not an ‘all or nothing’ attribute. Instead, motivation can be understood in terms of the degree to which it is experienced as autonomous (arising from within the individual) or controlled (imposed on the individual by an external regulator) [22]. Within Self-Determination Theory (SDT) [22,23], striving to be self-regulated or ‘autonomous’ is described as a basic human need and something pursued by individuals. Autonomy is defined as the extent to which a behaviour is experienced as internally generated, or self-determined [24]. SDT is a well-supported theory that has underpinned a body of research related to the purposeful goal striving of individuals in a range of contexts *e*.*g*., [25,26]. Goals and behaviours that are experienced as aligned to the values and beliefs of an individual are referred to as ‘self-concordant’ [25]. The self-concordance of goals has also been shown to predict goal success, commitment to the striving process and various aspects of wellbeing [27,28].

### Autonomy in controlled contexts

The human need for autonomy, and feeling that one is choosing to change behaviour, presents a specific challenge for implementation of evidence-based practices in the mental health field. Indeed, this issue emerges in any social context where there is a need to change the behaviour of individuals to comply with an overarching standard or set of social expectations [26,29,30]. There is evidence to suggest that socially controlling contexts are counter-productive to bringing about change, and can forestall implementation [26]. A central question is: How do we create autonomy for specific new practices (a sense that the change has come from within) when the change is imposed as part of the social context? We propose a structured, ongoing values-focused intervention for staff as one approach to enhancing autonomous motivation and therefore implementation of desired workplace change.

Values are defined here as verbal representations of desirable life consequences or ways of behaving that are enduring and pervasive across situations and contexts, [31] which can be enacted in moment-to-moment experience [32]. Values are widely viewed as important predictors and drivers of behaviour [33,34]. While the work-related goals or desired practices necessitated by a change initiative may be made explicit to staff, clarification of the values-base in which such goals are embedded is often overlooked in implementation efforts. By allowing mental health staff to connect with the values and intent of the change to practice, they are also able to identify how these overlay and potentially overlap with their own values as an individual and professional.

In such instances, though the change was not self-generated, it is possible that it will become more fully ‘owned’ as something that fits with the values and beliefs of individual staff members. This process is referred to as ‘integration’ [24], which has been described extensively within psychological and motivational literature [24,26]. Integration represents a shift from a controlled or imposed motivation for behaviour, to a more highly autonomous motivator for change [35]. When integrated, the motivation to act toward a specific goal or end-state is experienced as ‘arising from within’ due to its alignment with personally held values and beliefs. By attending explicitly to the values of staff, we propose an increased autonomous motivation for the desired workplace change and enhanced concordance between the ‘imposed’ practices and the values of individual staff, which will flow through to increased implementation.

**Figure 1:**
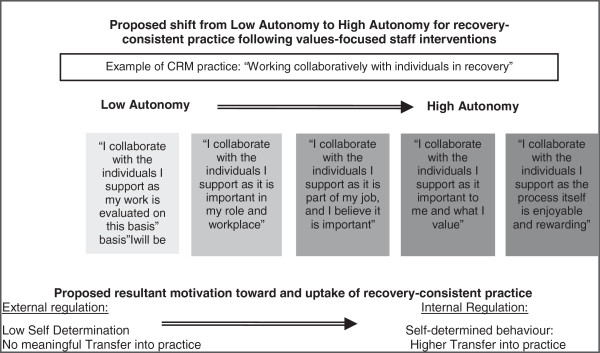
**Proposed increase in autonomy and implementation following values intervention.** This figure aims to illustrate the proposed mechanisms of change and outcomes hypothesised in this research.

Figure 1 demonstrates this proposed shift from low autonomy to a highly autonomous motivation for a key recovery practice, and the anticipated change in implementation:

### Aims and current research gaps

Autonomy is widely understood as an important factor in the purposeful striving of humans. Autonomy supportive practices are explored within education and developmental contexts [26,30], however there is an absence of research regarding the specific interventions to enhance autonomy in work contexts generally, and none that we are aware of in relation to mental health service delivery. Additionally, the autonomy supportive practices described in previous studies are not structured or standardised, and are therefore difficult to replicate or roll-out on a widespread basis. We propose a specific and structured set of interventions that actively promote clarification of and commitment to personally meaningful values of the mental health worker within the context of imposed organisational change.

## Methods

### Study design and procedure

This research is informed by two previous projects undertaken by this research team, and parallels data collection time frames to enable comparisons of implementation following the addition of the new intervention component. This project is supported by an Australian Research Council grant (LP09900708), with financial and in-kind contributions by the industry partners. Partner organisations are five well-established community-managed organisations involved in the direct provision of services to individuals living with severe and recurrent mental illnesses in the community. The partner organisations will nominate a number of suitable worksites drawn from across their service base to participate in the intervention and research components. We anticipate access to teams across a number of Australian states and government areas, allowing us to compare and control for the effects of geographical variables. In total, approximately 200 mental health workers from across 22 sites will be randomised and referred for intervention.

### Participants

Participants will be randomised by work-site to either the experimental (values) or control (implementation). Cluster-randomisation will be adopted to increase the feasibility of roll out in the organisational setting (*e*.*g*., consistency across what ‘change’ for individuals within a single worksite will look like) and minimise contamination across conditions (*i*.*e*., individual randomisation would likely lead to decreased fidelity to condition due to inevitable interactions between individual staff within teams). A computerised random integer program will be used to refer worksites to condition. Once randomised, staff from within sites will be referred for training and invited to consent to participate in the research process. Blinding will not be used at the participant or worksite level for pragmatic reasons (*i*.*e*., ongoing coaching will require condition-specific protocol) and to maximise fidelity (*i*.*e*., workers will consult with colleagues from worksites in the alternate condition during the course of duties and need to understand the importance of staying within assigned protocol). Information about previous exposure to the training (*i*.*e*., staff who have participated in some CRM training before time one) will be sought to allow screening prior to inclusion in the final data set. Staff who do not consent to participation in research but are within randomised sites will still participate in all intervention components to maximise the benefit of this project for the partners, to promote intervention fidelity within the workplace, and to allow all mental health workers access to contemporary evidence-based practices and techniques.

The intervention will be facilitated by our team and comprises both training (three days) and a 12-month coaching intervention. All teams will receive the same two days of training in the CRM [10]. On the third day of training, they will receive different training activities, according to the condition (values or implementation). In-service coaches (to be trained by our team) will conduct coaching. The in-service coaches will be supported with monthly, group-based coaching-support sessions facilitated by an appropriately skilled project member. Features of each intervention condition are as follows.

### Values condition

The values intervention is delivered as two components: Activities to support values clarification and commitment delivered on day three of training; and values-based coaching using CRM tools for 12 months within the workplace.

The aims of the values condition include:

1. Increase awareness of the values in which CRM is embedded.

2. Increase the extent to which the personal and professional values of the staff are explicit and expressed in the workplace.

3. Create opportunities for individual staff to identify the overlap or concordance of the CRM with their own values.

4. Regular and sustained (12 one-hour sessions each month) investment in the professional and personal development of staff via clarification and commitment to values-based goal-setting using CRM tools as the framework.

### Protocol for day three values intervention

The one-day values intervention is experiential in nature, using a structured values-clarification exercise with demonstrated utility in a range of clinical and non-clinical settings [36]. The purpose of this task is to help participants identify what values are most important to them, and to increase their awareness of the potential to actively pursue valued directions in both personal and professional domains of their life (*i*.*e*., increase the extent to which values are consciously used as a driver of purposeful behaviour). Staff members will be exposed to the concept of values in the standard CRM training (days one and two), and will have a basic theoretical and operational understanding of both the merit and applicability of working with values generally. Additionally, a focus on related concepts of willingness and commitment [37] that have been emphasised as important to values work will follow in day three.

Participants are given a set of 60 cards, each featuring a specific principle of living that is associated with a universal value-domain as outlined in Schwartz’ model [38,39]. They are then facilitated through a structured sorting task that titrates their focus down to the 15 valued directions each individual identifies as ‘most important to them.’

Following the card-sorting task, an additional intervention component is employed to foster intent and commitment to take purposeful steps toward valued areas of living. In this stage, the focus is on ‘life in general,’ and participants are asked to rate the extent to which they have purposefully been trying to enact a variety of values in the past 12 weeks. They rate their subjective success at moving toward each of the 15 specific valued directions identified as ‘most important.’ This process is structured around a worksheet based on the Personal Strivings methodology developed by Sheldon *et al*. that has been used extensively within the goal setting research [35,40].

Participants are facilitated through this process a second time, adopting a workplace focus. Participants are given the instruction; ‘conduct the card-sorting task again, this time focusing on what is most important to you in your current job.’ Following this, participants complete a second worksheet to rate the intent and self-reported success of recent striving toward the work values they have identified as most important at work.

The components thereafter focus on increasing awareness of the potential concordance between personal and professional values through a facilitated discussion session. Participants are invited to discuss commonalities between their ‘life in general’ list and their ‘workplace’ list. They are then asked to identify ways they can bring their ‘life in general’ principles into their workplace before being facilitated through the completion of the specific CRM values tool (known as the ‘camera’ [41]) as an initial commitment to this process.

### Protocol for values coaching

Individual coaching sessions adopt a structure known as the GROW model structure in both conditions. GROW was made popular by Graham Alexandar and John Whitmore and it is widely used in organisational and coaching contexts as a method of setting a basic frame to a coaching session [42]. It is particularly attractive in this case due its accessibility to those with little or no prior coaching experience. GROW is an acronym for the basic components of a coaching session—namely, goal, reality, options, wrap up/where to.

Individuals identified as suitable coaches within each partner organisations will be referred to a further half-day of coaching training conducted by the research team. Potential coaches are identified by managers within each of the organisations and also through a call for expressions of interest. Trained coaches are assigned to mental health workers within the same experimental condition, but outside line management to increase role clarity. Coaching consists of 12 hour-long sessions scheduled once per month and conducted in the course of paid working hours for both participants. The CRM tools are used as the framework for recording and structuring the recipients’ development across the coaching period, such that the participating staff members are using the tools that are part of the organisational change initiative themselves (*i*.*e*., in relation to their own values-based goals). The particular focus within the values condition is on the establishment of work-related goals that fit with the values stated by the recipient in initial training, and clarified as the coaching process continues.

### Implementation condition

The delivery of the implementation condition intervention components follow the same format as in the values condition but differ in focus and content. The day three of training in the implementation group is experiential in nature, but focuses on addressing opportunities for and barriers to the implementation of the CRM into practice within the workplace. The methodology used to structure and support the implementation intervention is the ‘Strengths, Weaknesses, Opportunities, and Threats (SWOT) Analysis’ developed by Albert Humphrey and used extensively in organisational contexts [43,44].

Coaching in the control condition is consistent in format and overall structure to the values condition. Implementation coaching adopts an alternate focus on identification and resolution of issues related to implementation of CRM in the workplace as identified by participants. For example, pragmatic issues (*e*.*g*., addressing technical issues associated with new practices) or attitudinal issues (*e*.*g*., working through resistance to change from clients or colleagues).

The aims of the implementation condition are as follows:

1. Ongoing exposure to and skills-based practice with the CRM.

2. Opportunity to identify and develop strategies to address factors that are impeding implementation (*e*.*g*., resistance from clients or co-workers).

3. Regular and sustained investment (12 x 1 month coaching sessions) in the professional and personal development of staff using CRM tools as the framework.

In both conditions, participants are using the same protocol and model of practice they are to use with their clients upon implementation. Coaching therefore promotes experiential learning [45] in both conditions. We expect this will result in increased transfer of CRM into practice compared to our previous research. In the values condition there is an additional parallel process such that mental health staff will be actively encouraged to work with the CRM practices in relation to their own lives and values, just as their clients would. That is, they are applying both the practices and the underlying processes ‘for real’ (values), rather than just practising in the use of the CRM (implementation). The value of parallel relationships in transferring knowledge from one dyad (*e*.*g*., supervisor and clinician) to another (*e*.*g*., clinician and client) has been elaborated within counselling literature [46]. We hypothesise this additional parallel relationship will increase implementation as a result of enhanced sense of meaning and connection with the CRM such that it becomes more internalised [47].

### Data collection and handling

The study will last 18 months with data collection at multiple times points. Primary data comprises a questionnaire battery completed by participants at five time points throughout the intervention (specific measures are outlined below). Data is also collected from coaches and recipients’ at each monthly coaching session to assess adherence to the GROW framework, integrity to experimental condition, and elements of the coaching alliance. In addition to self-reported measures of implementation, the study uses objective measures of transfer (also outlined below).

Data collection handling is in accordance with the procedures specified in the ethics approval obtained from the Human Research Ethics Committee at the University of Wollongong (HE09/221). A prime focus is on maintaining confidentiality of participants, which is promoted by the use of a unique self-generated identifier established at the first data collection point and re-used at subsequent collections. Additionally, because the research focuses on work-related variables and is being conducted in a work setting, individual data is forwarded to the research team directly (*i*.*e*., handed personally in sealed envelopes when on-site). This is to reduce possible biased responding and staff concerns that their individual information could be seen by superiors or other personnel within their organisation.

### Measures and data

The battery of measures will also include measures of intention to leave [48] job satisfaction [49], and burnout [50]. This additional data will be utilised by the research team to explore another set of hypotheses distinct from those being investigated here. Questionnaires pertaining to the outlined hypotheses are as follows.

### Staff knowledge and attitudes toward recovery

A range of questionnaires will be used to determine the knowledge, attitudes, and beliefs of participants related to the concept of recovery. These measures will be compared with previous research conducted by this team [51].

The Recovery Knowledge Inventory (RKI) [51] is a 20-item instrument that assesses mental health staff knowledge and attitudes about recovery using a five-point Likert scale. It has been used in previous research to assess pre-post change following intervention and has satisfactory psychometric properties [51].

The Staff Attitudes Towards Recovery Scale (STARS) is a 19-item measure developed and evaluated as part of the Crowe et al. study [52] and assesses attitudes and hopefulness related to the goal striving and recovery possibilities for clients. It has a five-point Likert response scale (strongly disagree to strongly agree) and higher scores reflect more hopeful attitudes. The STARS has satisfactory psychometric properties (α =.81) as established in previous work [52].

### Autonomy and values concordance

The Collaborative Recovery Model Values Questionnaire (CRM-VQ) is a modified version of the Personal Strivings questionnaire developed by Sheldon *et al*. [40]. The modification involves the use of perceived locus of causation from Sheldon’s strivings questionnaire, applied to the six components of CRM. This measure will examine the degree to which values embedded within the CRM are concordant with the personal values of participants. It will also assess the extent to which strivings toward the CRM practices are done for autonomous versus controlled (externally regulated) reasons.

### Self-reported transfer

#### CRM-VQ

This measure also includes items that assess the implementation intentions and self-reported success of acting purposefully toward the valued directions encompassed within the CRM. Items include ‘to what extent have you made specific plans about when, where, and how to put this value into play?’ and ‘In the last 12 weeks, I have been this successful in living this value,’ to which responses will be elicited on a five-point Likert scale. An additional item relating to anticipation of implementation barriers (‘to what extent have you anticipated possible distractions and obstacles to putting this value into play?’) has been included and will be rated in the same manner. This item will allow us to investigate the impact of deliberately addressing implementation barriers (control condition).

### Evidence of transfer within the workplace

Transfer indicators include time to implementation and maintenance of change. Transfer indicators will replicate those used previously by the research team [51]. We will seek direct evidence of implementation of any of the specific tools with the CRM (known as LifeJET) that are used to structure and record the recovery-focused practice of mental health workers. That is, examples of completed LifeJET documents within participant files. Time to implementation is calculated by determining the number of days lapsed between the date of training and the first date an example of completed LifeJET protocols was identified for participants. Maintenance of change is calculated by determining the proportion of staff work samples that are evidencing transfer (*i*.*e*., completed examples of CRM) after 12 months in comparison to time periods shortly after training (*e*.*g*., one month, six months).

### Objective audit of participant work samples for quality and overall transfer

A further objective audit is undertaken using an enhanced version of the Goal Instrument for Quality to determine whether principles of effective goal setting that underpin the CRM have transferred into practice following intervention. That is, the former transfer indicators ask ‘is the new practice being done, and to what extent?’ while this measure will enable exploration of the question ‘is there improvement in the quality of what is being done?’ This audit is conducted on-site by trained in-service assessors, and will allow investigation of the overall work practices of staff for evidence of changed practice. Client files will be randomly selected from three time-periods (*i*.*e*., zero to six months pre-intervention, zero to six months post-intervention, and six to twelve months post-intervention) from participants in each organisation. The care plans within files will be assessed on 17 elements of effective recovery-based goal setting by two in-service auditors. A copy of the de-identified file material is forwarded to the research team, who will also conduct the 17-point assessment of the care plan, enabling inter-rater reliability testing.

### Qualitative assessment of concordance between work samples and CRM values

A novel aspect of this research is the utilisation of a process-oriented protocol to assess worker adherence to the principles embedded within CRM. For example, the principle of personalisation (*i*.*e*., evidence of unique and person-centred approach to service delivery) is acknowledged as important in recovery [3]. Personalisation will be assessed across two dimensions (the content in the sample reflects unique expression of ideas; the language/presentation of content reflects individuality) using a three-point scale (0 = no, 1 = partially, 2 = yes). De-identified research copies of work samples are to be forwarded by participating mental health workers on a monthly basis by an on-site coordinator. Following power calculations, an online number generator will be used to facilitate random identification of an appropriate number of staff whose work-samples are to be reviewed. The samples of randomly identified staff will be reviewed using the six-point rating system at three time points (one month post-training, six months post-training, and 12 months post-training). Two trained assessors from within the research team will independently review the work samples for worker process and satisfactory inter-rater reliability will be established. We will compare observed adherence to CRM principles with the self-reported transfer and measures of autonomy obtained via the CRM-VQ (described above) at matching time periods (*i*.*e*., six and 12 months) during booster sessions to explore these differing elements of implementation.

### Data analysis

The intervention analyses will focus on two major questions: What aspects of Transfer does the values interventions positively influence? By what processes does the intervention work? We are primarily interested in investigating effects at the cluster-level, though will explore overall effects of the addition coaching-component on the entire sample and participant-level data in the qualitative processes described. The figure below presents a model of the analyses. Model A represents the total effect of values intervention versus control condition (implementation) (X) on Transfer (Y). Model B represents the direct effect of X on Y, and the indirect effect through the mediator (M), our psychological process variable (autonomy).

**Figure 2:**
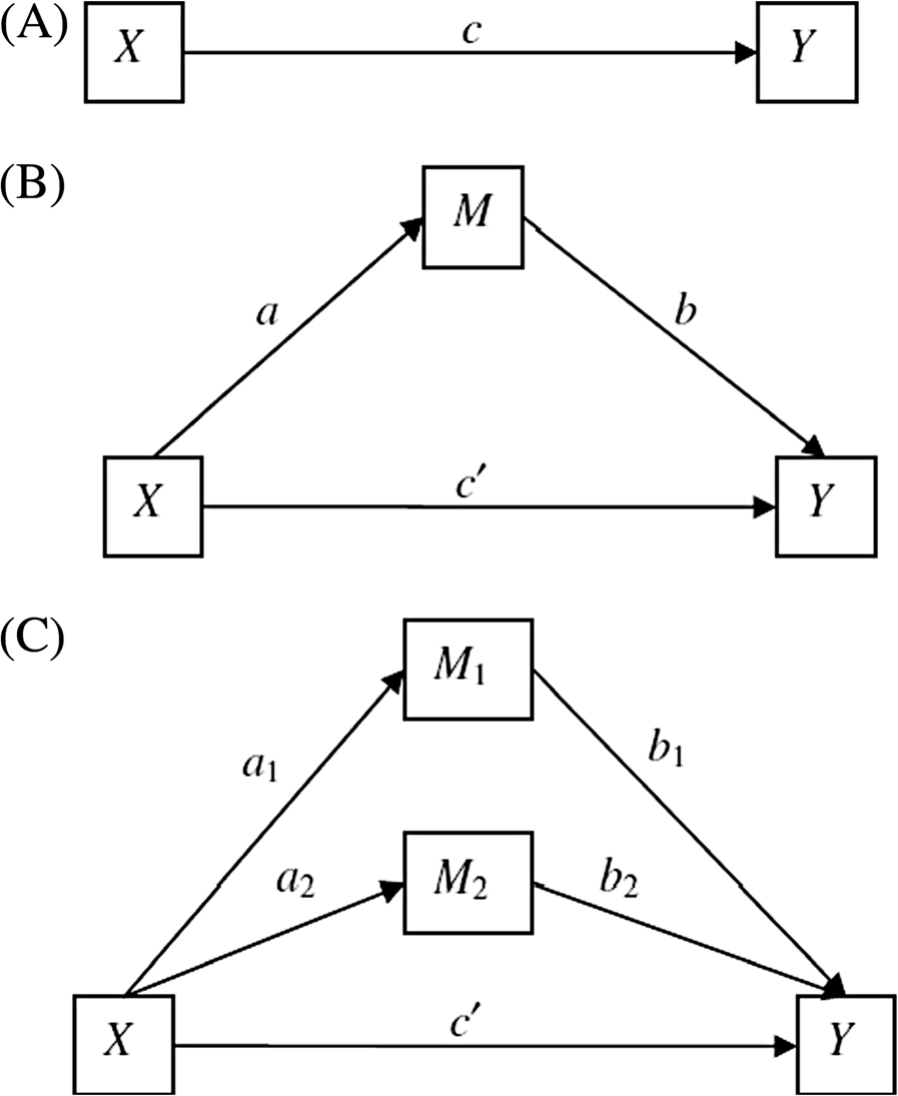
**Diagram of the hypothesised relationships between experimental variables.** This figure illustrates direct **(A)** and meditational **(B** and **C)** relationships between experimental (e.g. autonomous motivation) and dependant variables (e.g. implementation).

Model C is a multiple mediation model, and will allow us to test the extent that our intervention targets multiple process variables (*e*.*g*., value importance, value success, value commitment). Contemporary research in the area of autonomous motivation has begun to challenge the utility of aggregated measures as outlined above [*e*.*g*., 25]. Rather than being mutually exclusive or ‘either or’ constructs, it may be more relevant to investigate the particular elements of motivation as described on the continuum outlined above (Figure 2). This seems especially relevant in this case given the specific goals individuals are striving toward are not self-generated and occur in the workplace where ‘enjoyment’ is not necessarily a motivational force that is amenable or desirable as a target for change. So, we will also be able to test effects of various regulators (*i*.*e*., guilt, enjoyment, importance, fun) on the outcome variable. We will use the bootstrapping method described by Preacher and Hayes [53] to test the meditational models.

We will deal with missing data using full-information-maximum-likelihood estimation (FIML). Traditional approaches to missing data (*e*.*g*., list-wise or pair-wise deletion) can lead to considerable bias in parameter estimates. In contrast FIML provides a superior approach to dealing with missing data that uses all the available information for parameter estimation [54].

Standard multiple regression analysis will test for predictors of transfer. In terms of knowledge transfer related to the new workplace practices, we will compare current findings with the pre-post effect sizes extracted from the previous work conducted by the research team [51]. In that study, effect sizes were moderate based on Cohen’s criteria (STARS = 0.25, RKI = 0.52) with a sample size of 75. Given the additional components of this intervention we are expecting slightly higher effect sizes. Data collected in the CRM-VQ will enable comparison of these predictors with participant self-reported implementation of the new work practices.

Work-sampling audits will also be used as an objective measure of transfer. We are anticipating a sample size of 200 at time point one, but have allowed for a more modest sample size of 100 in calculating power analyses. In our previous study, we found 37% of people transferred training. With this information we are able to estimate the percentage of people who need to demonstrate transferred practices in work samples in order to show a significant increase in the proportions between the former and present studies. Using Z test for proportions we have calculated that we need at least 48 out of the 100 participants to obtain a significant difference at p<0.01 to detect a 10% increase in transfer. We believe this difference is very achievable with the addition of the values-focused interventions.

## Discussion

### Anticipated challenges

There are a number of challenges to carrying out this project due to the applied and organisational nature of this research. The schedule of intervention needs to meet standards of feasibility and pragmatism for the partner organisations, which are contributing significantly in terms of in-kind and cash contributions. Such challenges include the need to roll-out intervention components at a rate that enables partners (who are service providers) to equip and up-skill staff and accommodate the needs of new staff, which will at times put pressure on the capacity of the research team to deliver the intervention. Additionally, the need for interventions (particularly coaching) be practical and manageable has influenced the choice of methodology to be employed (*e*.*g*., GROW method to structure coaching interactions). A prime focus for the partner organisations and the research team has been ongoing sustainability of the interventions beyond the formal support of this project. This has necessitated the interventions be amenable to being ‘passed on’ to in-service champions in a train-the-trainer model, for example.

There is risk of data loss due to the multiple time points and staff turnover rates within the mental health field. Turnover of staff in mental health service organisations is up to 26% per annum [55]. These risks are being managed by allocation of a designated project coordinator within the research team, who will provide day-to-day liaison with the industry partners and take oversight of the intervention schedules and data collection. Each industry partner will identify a liaison officer who will have carriage of the coordination and scheduling responsibilities within their organisation. Our relatively large sample size anticipated for time pointone has been estimated with consideration given to the aforementioned industry attrition rate.

A further challenge in this research is to maximise fidelity to condition in this project. As stated above, cluster randomisation by worksite was adopted as a primary means of reducing contamination and enhancing utility and effectiveness of the rollout within the workplace. It is not possible to blind staff to condition, and it is likely there will be a degree of contamination as staff discuss the changes to work practice with colleagues from other sites (*e*.*g*., at training days or meetings). Participants in each condition will be aware that their colleagues may be receiving different components of training in the other condition. To address commitment to condition, both groups receive a strong rationale for the training and coaching approach they receive. Consistency to condition will be monitored specifically via the coaching record form completed alongside monthly coaching sessions. A ‘lessons learned’ and ‘risk register’ will be kept to manage these challenges, and are important to the ongoing development of the implementation literature.

## Conclusion

Training continues to be a popular method used in workforce development, yet the problem of inadequate transfer continues [56]. With reported annual training investments exceeding $50 billion in large economies like the United States [57], even modest increases in the return on investment is highly desirable. This research has wider significance to all workforces in terms of understanding the factors that influence and promote uptake of organisational change initiatives. To our knowledge, there are no other research studies in the organisational context that employ specific values-based protocol as a means to enhancing worker autonomy for and uptake of desired practices. Indeed, in the area of mental health, ‘effective transfer’ has benefits, including optimal provision of services to those within a vulnerable population, that may be argued as more important than the significant fiscal advantages outlined above. A key priority of many recent policy statements of governments across Europe and English-speaking economies is delivery of recovery-oriented service [58,59]. This research aims to directly address this priority area. While not elaborated here, we foresee cumulative benefits to mental health service participants, staff, and organisations as a result of the impacts of this specific intervention on employee satisfaction and wellbeing. These results have been demonstrated in previous studies investigating self-concordance of goals [27,28].

While problems of implementation are being acknowledged more widely, there is still uncertainty as to how to operationalise and measure successful transfer [60,61]. In particular, the role of values as a means to promoting uptake and a construct to be measured within the science of implementation requires elaboration. A range of models and measures exist to explain and capture uptake of new practices, ranging from attitudinal [62] to supervisor-rated [63], objective measures of observed performance on the job [64], and composite measures that combine multiple elements [65]. While the latter of these allow a snapshot of whether a desired practice is being carried out within the workplace, they do not allow us to make any assertions about ‘how’ the work is being done.

Our study explores the relevance of values in promoting ‘role-extra’ behaviours that represent enactment of embedded principles within implementation initiatives. The relevance of working from a values base and enabling staff involved in a change-initiative to connect with the principles embedded within it, is emphasised in the field of mental health recovery [66]. The important and little-explored issue of measuring adherence to the values in which a desired practice is embedded will be investigated further in this research.

## Competing interests

The authors declare there are no competing interests.

## Authors’ contributions

VW developed and prepared this manuscript as part of her PhD and has a figural role in the implementation of intervention components. VW will be primarily responsible for analysis and reporting of research findings. She has contributed to conceptual development of this project particularly in relation to the measurement of value-congruent implementation. LO is a chief investigator in this project, primary supervisor of the PhD candidate and contributed to the writing of this manuscript. FD is lead researcher on this project and was responsible for the coordination of the grant application and other key elements of project development. FD will maintain responsibility for coordination for overall project, data and publications. FD has inputted and commented on this manuscript. TC will act as coordinator of the intervention roll out and is also a chief investigator on this project. TC has commented on this manuscript. JC is a chief investigator on this project and will provide specific input regarding the analysis plan. He provided comment on the manuscript. RA will be responsible for data management and logistical oversight of the project. All authors read and approved the final manuscript.
